# Acute vision loss in post-partum period as presenting symptom of HIV-associated cryptococcal meningitis–an unusual case report

**DOI:** 10.1186/s12879-016-1925-0

**Published:** 2016-10-19

**Authors:** Aniruddha More, Ravindra Kumar Garg, Hardeep Singh Malhotra, Neeraj Kumar, Ravi Uniyal

**Affiliations:** Department of Neurology, King George Medical University, Lucknow, PIN-226003 Uttar Pradesh India

**Keywords:** Acquired immune deficiency syndrome, Human immunodeficiency virus, Meningitis, Optic nerve

## Abstract

**Background:**

Acute vision loss in the post-partum period can occur due to many reasons. Eclampsia, posterior reversible encephalopathy syndrome (PRES), pituitary apoplexy, and central serous retinopathy are some of the important causes. Cryptococcal meningitis as a cause of acute vision loss in the post-partum period has not been mentioned in literature.

**Case presentation:**

A 25-year-old female presented to us with acute bilateral complete vision loss in the post-partum period. Her serum was tested positive for HIV antibodies. Cerebrospinal fluid (CSF) examination revealed cryptococcal meningitis. She was started on amphotericin B, antiretroviral drugs, and steroids. Though symptoms of meningitis resolved after treatment no significant improvement in vision was observed at 3 months.

**Conclusions:**

Cryptococcal meningitis may be considered as one of the causes of acute vision loss in pregnant/post-partum females with human immunodeficiency virus (HIV) positivity.

## Background

Approximately, 36.7 million people globally were suffering from HIV infection at end of 2015 [1]. About 17.4 million women were suffering from HIV infection worldwide in 2014 [2]. HIV infection in pregnancy has become the most common complication of pregnancy in some developing countries. In pregnancy immune function is suppressed in HIV infected as well as non-infected subjects [3]. There is a reduction in immunoglobulin, reduced complement levels and a significant decrease in cell-mediated immunity during pregnancy [3]. This leads to increased susceptibility of pregnant females to opportunistic infections like tuberculosis and cryptococcal meningitis. Cryptococcal disease is very uncommonly seen in HIV infected pregnant females and to date only three cases of HIV infection with cryptococcal meningitis have been reported during the peripartum period [4]. Symptoms of cryptococcal meningitis include fever, headache, nausea/vomiting, altered sensorium, and multiple cranial nerve palsies. Vision loss is seen in 33–47 % of patients [5]. Seven percent of HIV infected individuals suffer from cryptococcal meningitis in the course of their illness out of which only 1.9 % have cryptococcal meningitis as the presenting symptom [5, 6]. Complete bilateral vision loss as presenting symptom of HIV related cryptococcal meningitis is extremely rare [7]. Similar presentations in post-partum female, as it was in our case, are unique and high index of suspicion is necessary for accurate diagnosis.

## Case presentation

**Fig. 1:**
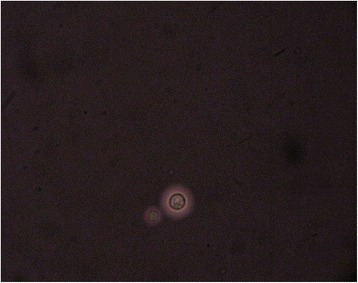
India ink preparation showing capsules of *Cryptococcus neoformans*

**Fig. 2:**
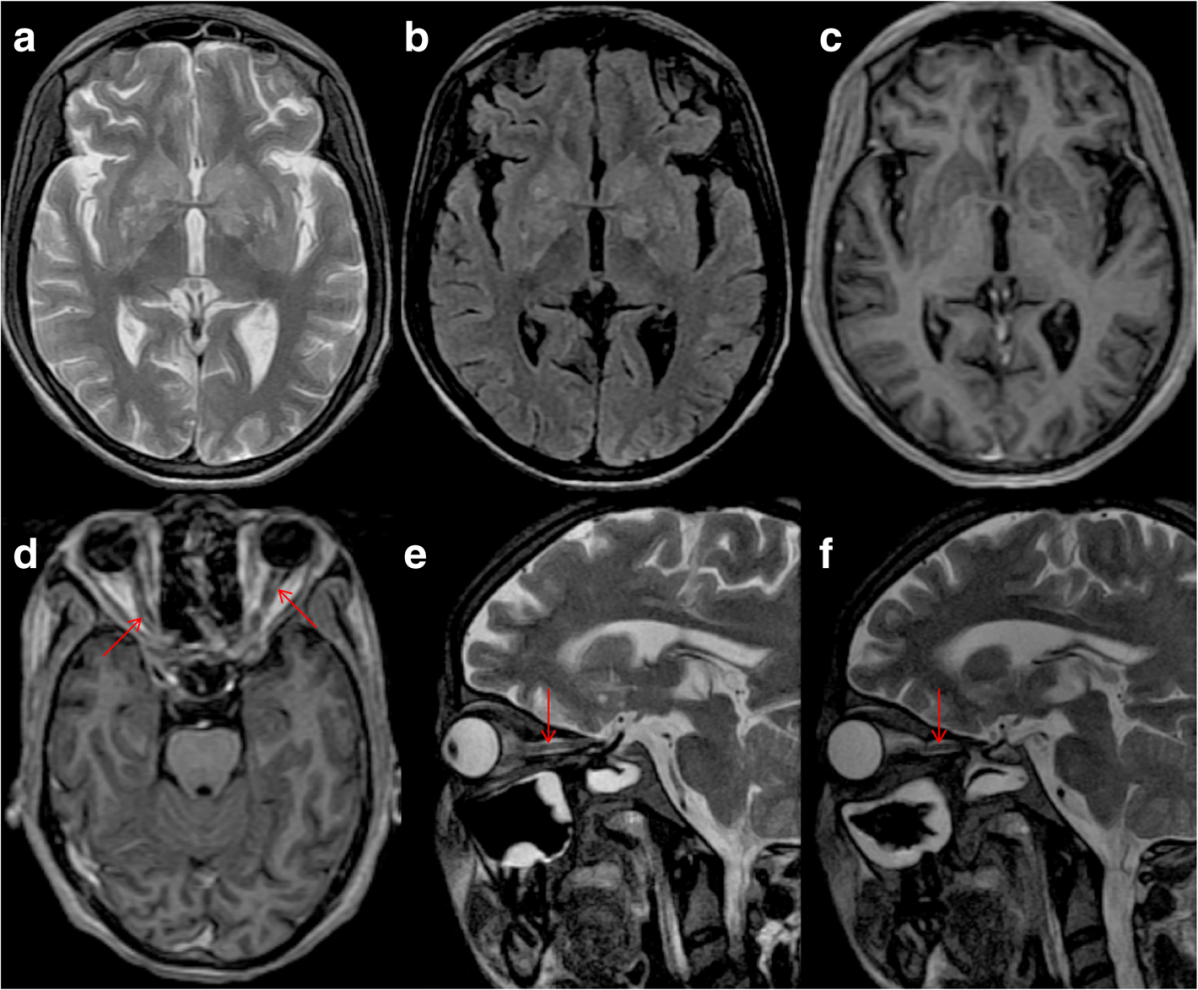
MRI of the brain depicts hyperintense signal intensity changes in the basal ganglia on axial T2-weighted (**a**) and FLAIR (**b**) sequences. There is no suggestion of contrast uptake in the basal ganglia (**c**) while both optic nerves demonstrate enhancement (**d**) on SPGR-GAD sequence. T2-weighted para-sagittal orbital sections show hyperintensity in the right (**e**) and the left (**f**) optic nerves (arrows)

A 25- year-old previously healthy pregnant female in 36th week of her gestation presented in labour to the department of Obstetrics & Gynecology of our hospital. Before admission she was having mild to moderate headache of 10 days duration which was associated with mild fever with intermittent high grade spikes which used to subside on administration of antipyretic medications. Her serum tested positive, by ELISA, for antibodies towards HIV. She delivered a still-born baby vaginally. She was apparently asymptomatic for 2 days, besides fever and headache which were relieved by antipyretics and analgesics. On the 3rd day post-partum day, she woke up in the morning realizing that she was not able to see from both eyes; the loss of vision was painless and symmetrical. She was transferred to the department of Neurology unit of our hospital for further evaluation. On clinical examination, her vitals and level of consciousness were normal. Her visual acuity was significantly reduced and she could only perceive light in both eyes. Her pupils were completely dilated and not reacting to light. Fundus examination revealed bilateral edematous optic discs suggestive of papillitis. Other cranial nerves were normal on examination. Motor and sensory system examination were normal. Plantar reflex was bilaterally flexor. She had neck stiffness and Kerning’s sign was positive. Urgently a CT scan of her head was done which showed no significant abnormality and then her CSF examination was done based on her clinical signs. Her CSF opening pressure was mildly elevated (200cms of H_2_O). CSF examination showed mild pleocytosis (35 cells/mm^3^) with lymphocytic predominance, mildly elevated proteins (76.5 mg%) and low sugars (32.7 mg% with corresponding blood glucose of 117 mg%). Her CSF was tested positive for Cryptococcus by India ink staining (Fig. 1). The CSF showed a titre of 1:100 for cryptococcal antigen by latex agglutination method. Since she was diagnosed with cryptococcal meningitis she was categorized as having stage 4 disease according to WHO clinical staging for HIV/AIDS. All other hematological and biochemical parameters were within normal limits. Her CD4 counts were done which turned out to be 166 cells/mm^3^. The plasma HIV-RNA levels were not performed in this case. As per National AIDS Control Guidelines of India prior HIV-RNA levels assessment, before starting antiretroviral drugs, is not mandatory. Her MRI was planned to evaluate the cause of her blindness which showed signal intensity changes in bilateral optic nerve and (cryptococcoma/cryptococcal) infiltration in bilateral ganglio-capsular region (soap-bubble appearance) (Fig. 2). She was initiated on amphotericin-B 0.7 mg/kg/day IV once daily (slow infusion) and intravenous methylprednisolone 1 gm IV once daily (for 5 days), and an antiretroviral regimen consisting of zidovudine (300 mg once daily), lamivudine (150 mg once daily), and nevirapine (200 mg once daily) was planned. She received amphotericin B for 21 days after which she was started on tablet fluconazole 400 mg per day which was continued for 8 weeks. Antiretroviral medications were started after completion of intravenous antifungal medications. Her headache improved with treatment and she became afebrile. Repeat CSF examination was negative for Cryptococci by India ink staining. Though she showed significant improvement in meningitic symptoms, her vision did not show any significant improvement even at end of 3 months of her illness.

## Discussion

Acute vision loss in the post-partum period is reported due to severe preeclampsia/eclampsia (in which vision loss could be due to exudative retinal detachment, hypertensive retinopathy and cortical blindness) [8], PRES which can cause reversible acute vision loss [9], pituitary apoplexy [10], posterior ischemic optic neuropathy [11], anterior ischemic optic neuropathy [12], cortical venous sinus thrombosis [13], and central serous retinopathy [14]. Cryptococcal meningitis as a cause of acute vision loss in the post-partum period has not yet been reported in literature.

Vision loss in HIV infected individuals can be due to infectious and non-infectious causes [15–17]. HIV retinopathy is the most common non-infectious ocular complication. Amongst infectious causes cytomegalovirus retinitis is considered as the most common ocular infection. Neuro-ophthalmic lesions affected 6 % of individuals of acquired immunodeficiency states of which most common cause was cryptococcal meningitis. Approximately 25 to 50 % patients with cryptococcal meningitis demonstrate a neuro-ophthalmic complication. In a cross-sectional analysis looking at the ophthalmic complications in the pre-HAART (Highly Active Anti-Retroviral Therapy) and HAART period, it has been suggested that despite of an advancement in terms of treatment the difference between the two treatment protocols has not been significant (pre-HAART (41.67 %) versus HAART (38 %). Of significance was the fact that the odds of development of ophthalmic complications with a CD4 count of >200 cells/μL were significantly higher (4.87) during the HAART period [17]. Since most patients with ophthalmic complications have been noted to present in WHO stage 3 and 4, it has been recommended that regular ophthalmic assessments are done in patients with HIV positivity.

Cryptococcal meningitis is one of potentially fatal opportunistic infection seen in HIV infected individuals. The causative agent is *Cryptococcus neoformans*, an encapsulated fungus commonly isolated from pigeon droppings. Humans are infected by inhalation of organism [18]. Human to human transmission is yet to be documented [19]. Signs and symptoms of cryptococcal meningitis include headache (80–92 %), meningeal signs (50–80 %), nausea/vomiting (40–80 %), fever (36–67 %), cranial nerve palsies (28–60 %), vision disturbances (33–47 %), confusion/somnolence (33–40 %), ataxia/gait disturbance (26–40 %), papilledema (28–33 %), seizures (13–15 %) [5].

Vision loss in cryptococcal meningitis can be rapid (<3 days) occurring as rapidly as 12 h or can occur slowly (>3 days) over weeks to months [20]. Possible mechanisms of rapid visual loss include direct invasion of optic nerve by Cryptococcus, optic neuritis, and optochiasmatic arachnoiditis while a possible mechanism for slow vision loss has been attributed to raised intracranial pressure [20]. Factors predicting vision loss are presence of papilledema, high CSF opening pressure, and a positive India ink preparation. In cases with acute vision loss despite of giving antifungals and steroids no substantial improvement in vision is expected and these cases tend to have a poor prognosis for visual recovery. In cases with slow visual loss therapeutic measures to reduce intracranial pressure like shunting or optic nerve sheath fenestration have been shown to halt vision loss and shown improvement in some cases [20].

Vision loss in our patient was most probably due to infiltration of optic nerve by Cryptococcus causing optic neuritis and acute vision loss. This was supported by fact that her MRI showed enhancement of both optic nerves and presence of papillitis on ophthalmoscopy with cryptococcal infiltration in both ganglio-capsular regions. This also explains her poor recovery of vision despite of receiving steroids as noted in previous studies (Table 1). The general belief that pregnancy is a state of immune suppression may not hold true in all patients. Pregnancy, indeed, is a state of an altered immune status which may respond to different stimuli variably. Thus, in the background of pregnancy and a HIV positive state, this rare manifestation of Cryptococcus appears to have evolved [21].

**Table 1:** A review of published cases of vision loss in Cryptococcal meningitis (Cases, reported after year 2000 and those who had a MRI done, were included)

Reference	HIV status	Summary of case	MRI	Suggested cause of vision loss	Outcome
Ng et al. [22]	Positive	A 39-year-old Chinese man presented with bitemporal headache, giddiness and vomiting over a period of 2 days. There was no Cryptococcus detected by Indian ink examination of the CSF but CSF culture grew Cryptococcus neoformans. The patient was treated with amphotericin, and then to fluconazole.	Normal	increased intracranial pressure	irreversible blindness in both eyes
	Positive	A 36-year-old Chinese man presented with subacute onset of severe headache and confusional state. CSF microscopy with Indian ink examination showed Cryptococcus. Eight days after starting IV amphotericin, he complained of bilateral blurring of vision and had a seizure. A repeat lumbar puncture showed raised opening CSF pressure of more than 40 cm and 20 mL of CSF was withdrawn. During follow up, concentric diminution of visual field in both eyes was recorded. A lumbo-peritoneal shunt was done.	CT was normal	Increased intracranial pressure	Normal vision
Mohan et al. [23]	Positive	A 41-year-old male with a history of cryptococcal meningitis admitted with severe headache. On the 6th day, He had complete loss of hearing, vision loss as well as bilateral facial palsy and bilateral sixth nerve palsy. Bilateral papilledema was seen. CSF opening pressure was 460 mm H2O. Amphotericin and Flucytosine were given. Repeated lumbar punctures were done but pressure remained over 500 mm H2O. Ommaya reservoir placed. There was dramatic clinical improvement of the patient.	Hydrocephalus	Increased intracranial pressure	Regained his vision partially
Hong et al. [7]	Positive	A 58-year-old man presented with acute vision loss. Patient had normal CSF opening pressure and fundus. He received antiretroviral and antifungal agents.	Normal	Possibly, direct fungal infiltration of the optic nerve, optic chiasm, or optic tracts	Vision improved
Milman et al. [24]	Positive	25-year-old patient developed headaches, seizures, altered mental status, and visual loss. Lumbar puncture showed markedly increased opening CSF pressure; cryptococcal organisms were identified by India ink preparation and in culture. Despite treatment with amphotericin and fluconazole, visual loss progressed.	leptomeningeal and optic nerve enhancement without hydrocephalus	increased intracranial pressure	Improved with bilateral optic nerve sheath fenestration.
Muslikhan et al. [25]	Negative	A 17-year-old boy presented with blurring of vision in both eyes and diplopia for 3 weeks. Extraocular muscles movement showed bilateral sixth nerve palsies. Discs were hyperaemic and slightly elevated. Lumbar puncture revealed high opening pressure >300 mmH(2)O. CSF showed *Cryptococcus neoformans*. IV amphotericin and fluconazole were given.	CT scan of the brain was normal	high intracranial pressure	His vision was improved to 6/6 in both eyes with recovery of peripheral visual field.
De Socio et al. [26]	Positive	A 32-year-old man with disseminated Cryptococcosis was being treated with antiretroviral therapy. On day 7 he had a unilateral vision loss.	retrobulbar neuritis	IRIS leading to optic nerve neuritis following anti-retroviral therapy	At 3 months, vision was normal After starting ART and IV methyl-prednisolone
Duggan and Walls [27]	Positive	A 39-year-old man with AIDS presented with recent onset of headache, dizziness, and syncope. CSF showed *C neoformans*. Amphotericin and flucytosine were started. On day 2, the patient had several episodes of complete loss of vision bilaterally. Drainage of CSF to decrease ICP resulted in the immediate return of vision. External ventricular drain was placed and later optic nerve sheath fenestration.	optic nerve edema or neuritis	high intracranial pressure	permanent vision loss
	Positive	A 39-year-old African female with AIDS presented with headache, neck pain, and altered mental status of 3 days. CSF showed *C neoformans*. On hospital day 2, the patient complained of sudden complete loss of vision bilaterally. Retinal microvasculopathy was noted.	Normal	Retinal microvasculopathy	Patient died
Espino Barros Palau et al. [28]	Out of three cases one was HIV positive	A 46-year-old male had bilateral optic atrophy. He had been well until 3 months prior when he experienced vision loss, headache, nausea, and fever.	Normal	Intracranial hypertension	serial lumbar drain
		A 43-year-old female presented with 3 weeks of headache, horizontal diplopia, and bilateral vision loss. Patient had renal transplantation requiring immunosuppression in 2005. CSF opening pressure was markedly elevated. Amphotericin B and flucytosine were initiated.	Diffuse leptomeningeal enhancement and punctate areas of enhancement in the pons and basal ganglia.		
		A 35-year-old male presented with 1 month of bilateral progressive vision loss. Ophthalmoscopy showed peripapillary subretinal fluid extending into the macula bilaterally. Both optic discs had edema. CSF opening pressure was elevated. Amphotericin B and flucytosine were started.	Multiple T2-hyperintense lesions throughout the cerebral hemispheres, brainstem, and cerebellum as well as with leptomeningeal enhancement.		
Portelinha et al. [29]	Negative	A 52-year-old woman with headaches, vomiting and fatigue for 3 weeks. She was diagnosed with cryptococcal meningitis and treated with antifungal therapy. She also had decreased vision in the left eye, bilateral sixth nerve palsy and papilloedema. A ventriculo-peritoneal shunt was placed and methylprednisolone was started.	Signs of intracranial hypertension as well as multiple parenchymal lesions and optic nerve sheath enhancement.	high intracranial pressure and optic nerve fungal infiltration	In spite of the control of intracranial pressure there was a decrease in vision in the right eye and deterioration of visual fields.
Ghatalia et al. [30]	Positive	A 34 year old man of vision loss in a patient with Cryptococcal meningitis and normal ICP.	Abnormal circumferential enhancement within the bilateral optic nerve sheaths.	IRIS leading to optic nerve neuritis	Vision improved with corticosteroids
Merkler et al. [31]	Negative	A 38-year-old woman presented with bilateral vision loss. Empiric steroids resulted in improvement in visual acuity, while tapering steroids, she had visual loss again.	Multiple areas of ill-defined enhancement in the optic chiasm and tracts.	Invasion of the optic apparatus by *Cryptococcus neoformans*	One year later, symptom free.

## Conclusions

Cryptococcal meningitis may be considered as one of causes of acute vision loss in HIV-infected pregnant females with suggestive symptoms; needless to say that a high index of suspicion should be kept to diagnose the condition. Though prognosis of visual recovery is poor in cases of acute vision loss, early therapeutic measures to relieve intracranial pressure may potentially halt the progression while some recovery may be observed in cases with slowly progressive vision loss.

## Abbreviations

AIDS: Acquired immune deficiency syndrome
ART: Antiretroviral therapy
CSF: Cerebrospinal fluid
CT: Computed tomography
HAART: Highly Active Anti-Retroviral Therapy
HIV: Human immunodeficiency virus
ICP: Intracranial pressure
IRIS: Immune Reconstitution Inflammatory Syndrome
IV: Intravenous
MRI: Magnetic Resonance Imaging
PRES: Posterior reversible encephalopathy syndrome
